# Multimodal preclinical study of long-term effects of prenatal ultrasound on the mouse central nervous system

**DOI:** 10.1038/s41598-026-49034-9

**Published:** 2026-04-21

**Authors:** Peter Karolyi, Zita Kepes, Bence Pelyvas, Maria Marosi, Bela Juhasz, Ervin Berenyi, Angelika Englohner, Elizabet Kocsi, Beata Pelles-Tasko, Alejandro Trouvé-Carpena, Silvia De Santis, Santiago Canals, Gyorgy Trencsenyi, Zoltan Meszar, Tamas Papp, Péter Károlyi

**Affiliations:** 1 Department of Radiology and Imaging Science, Institute of Medical Imaging, Faculty of Medicine, University of Debrecen, Nagyerdei St. 98, Debrecen, H-4032 Hungary; 2 Gyula Petrányi Doctoral School of Clinical Immunology and Allergology, Faculty of Medicine, University of Debrecen, Nagyerdei St. 98, Debrecen, H-4032 Hungary; 3 Doctoral School of Neuroscience, Faculty of Medicine, University of Debrecen, Nagyerdei St. 98, Debrecen, H-4032 Hungary; 4 Department of Nuclear Medicine and Translational Imaging, Institute of Medical Imaging, Faculty of Medicine, University of Debrecen, Nagyerdei St. 98, Debrecen, H-4032 Hungary; 5 Department of Pharmacology and Pharmacotherapy, Faculty of Medicine, University of Debrecen, Nagyerdei St. 98, Debrecen, H-4032 Hungary; 6 Department of Emergency Medicine, University of Debrecen Clinical Centre, Health Care Service Units, Clinics, University of Debrecen, Nagyerdei St. 98, Debrecen, H-4032 Hungary; 7 Instituto de Neurociencias, Consejo Superior de Investigaciones Científicas, Universidad Miguel Hernández, Sant Joan d’Alacant, Alicante, Spain; 8 Department of Anatomy, Histology and Embryology, Faculty of Medicine, University of Debrecen, Debrecen, 4032 Hungary

**Keywords:** Cognition, Diffusion tensor imaging (DTI), Hippocampus, Memory, Neurodevelopment, Positron emission tomography (PET), RNA sequencing, Ultrasound, Developmental biology, Neuroscience

## Abstract

The development of the central nervous system is regulated by multiple spatio-temporally varying factors that are essential for neurons to form functional networks. Prenatal ultrasound (US) exposure may affect the dendritic arborization and spine density of CA1 neurons in the hippocampus—a key region for memory processing—but the mid- and long-term effects of this exposure are still not well understood. In this study, we used an animal model to examine how repeated prenatal US exposure impacts memory function. After treatments, we assessed the memory performance of both treated and control animals and analysed the CNS for microstructural changes in the brain tissue. We also explored potential changes in glucose metabolism and hippocampal transcriptomic profiles, focusing on calcium signaling. Our results did not reveal any changes in memory performance in US-exposed animals, despite notable microstructural impairments detected in several white matter tracts. RNA sequencing analysis revealed that US exposure causes lasting changes in calcium signaling pathways, which are known to be crucial for memory formation. Overall, our findings demonstrate that prenatal US screening in an animal model can produce enduring molecular, structural effects, underscoring the importance of further research into the safety and long-term implications of prenatal US screening.

## Introduction

Prenatal ultrasound (US) is a widely accepted and routinely used diagnostic tool worldwide for visualizing the fetus and fetal anatomy. Since there is no evidence of harm to either the fetus or the mother, multiple US examinations can be safely performed during pregnancy to monitor fetal development and identify any potential structural abnormalities^1^. To protect patients, the U.S. Food and Drug Administration (FDA) has set the maximum allowable spatial-peak temporal average intensity for US transducers at 720 mW/cm^2^. Additionally, the ALARA (As Low As Reasonably Achievable) principle is critical for safety regulations^2–5^.

Beyond these regulations, other parameters related to the bioeffects of US exposure, such as the mechanical index (MI) and the thermal index (TI), are also routinely monitored during examinations. The MI indicates the potential of the US beam to cause cavitation, while the TI signals the risk of temperature increase in soft tissues. Both indices are expected to stay below 1. However, some protocols allow for higher MI and TI values, as long as the maximum duration of scanning or testing is predefined^4^. For documentation purposes, both MI and TI are always shown on the US machine screen.

Another important aspect, according to recent research, is that a 10-minute US screening using parameters typical for human diagnostics (10 min, MI and TI below 1, FR: 3 MHz), leads to elevated levels of c-fos expression and a subsequent increase in the release of brain-derived neurotrophic factor (BDNF) protein^6,7^. Given the critical role of BDNF in dendritic growth, spine maturation, and the genesis as well as formation of spines, its ultrasound-triggered elevation may exert a significant impact on the central nervous system.

Previous studies have clearly demonstrated the association between US stimulus, BDNF expression, and spine morphology^6,8^. For example, in a preclinical study by Tyler and Pozzo-Miller et al., animals exposed to US exposure exhibited significantly higher spine density and enlarged spine volume in the CA1 region of the hippocampus, which was linked to elevated BDNF levels^8^. Dendritic spines have a major contribution to synaptic transmission, and their dynamic changes in number, size, and shape during development and learning processes are closely correlated with the strength of synaptic connections^9^.

Furthermore, spines are also linked to long-term potentiation (LTP) and long-term depression (LTD)^9–12^. Thin, smaller spines usually indicate an immature state, while larger, more stable spines are characterized by higher postsynaptic densities and greater volumes^9,13^. This is supported by a loss-of-function BDNF study, where using BDNF scavenging antibodies on primary hippocampal neurons led to a decrease in dendritic spine head width and an increase in spine length, indicating a less mature phenotype^14^.

Although the role of BDNF in learning and memory remains somewhat controversial, human studies have shown a positive correlation between serum BDNF levels and cognitive function in elderly patients^15,16^, while other researchers have reported conflicting results^17–19^.

Pyramidal CA1 neurons play a crucial role in memory formation and are highly responsive to BDNF^14–19^. Diagnostic levels of US can affect CA1 neurons by increasing spine density and maximum spine diameter on the basal dendritic tree, as well as elongating dendrites and increasing the average length of dendritic segments^7^. Interestingly, no study has yet reported the effects of US exposure on the apical dendritic tree^7^. Furthermore, previous results suggest that US treatment can prevent dendritic loss in CA1 neurons and inhibit atrophy in the hippocampal complex^20^. Additionally, US exposure has been shown to modulate theta oscillations, which are essential for memory formation^21–26^.

Considering all this, we aimed to explore the potential effects of repeated US exposure on memory function at the preclinical level. We used various imaging techniques, including positron emission tomography (PET) and diffusion-weighted magnetic resonance imaging (MRI), along with behavioral testing via the Morris Water Maze (MWM) and RNAseq analysis. We found that repeated US exposure in mice can produce lasting molecular, structural, and cognitive effects, underscoring the need for further research into its safety and long-term implications.

## Materials and methods

### Animal housing and breeding

**Fig. 1:**
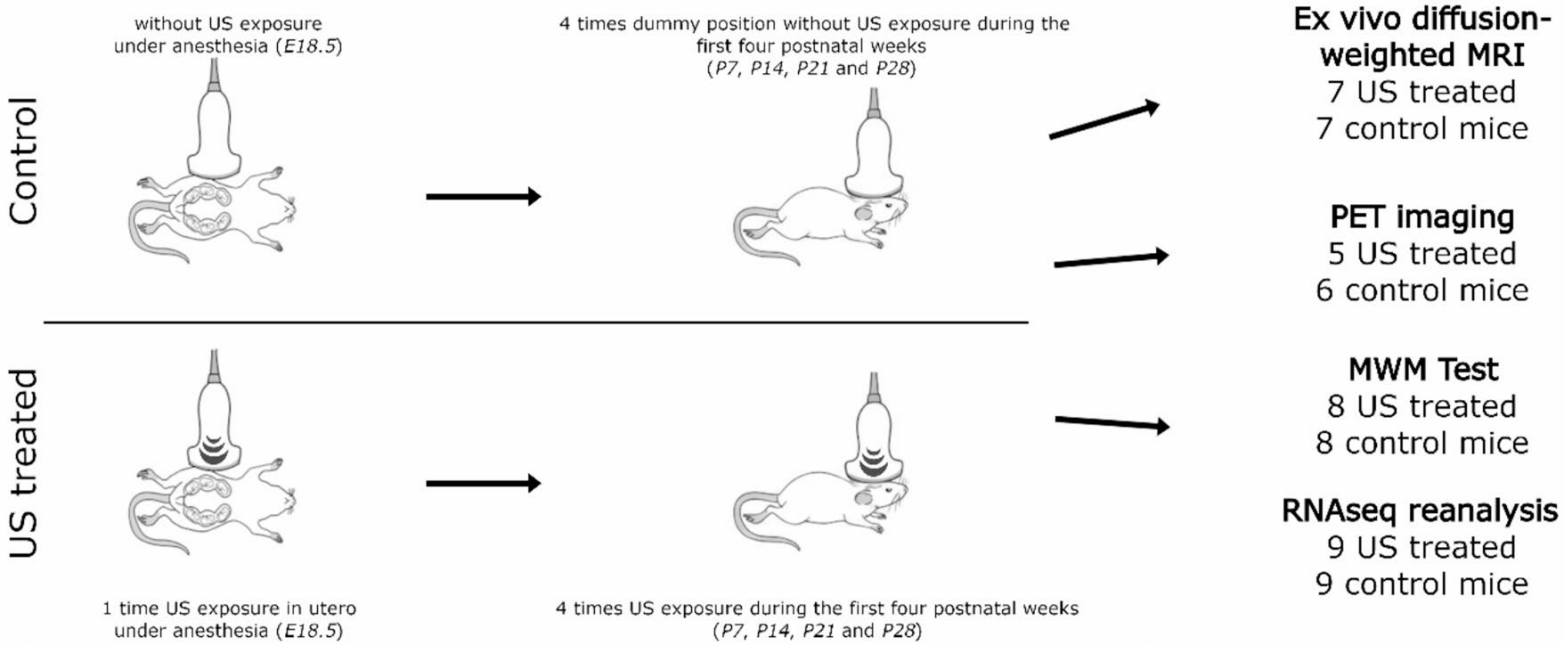
Schematic illustration of the experimental design employed in this study. The embryos were either exposed to ultrasound stimulation or underwent sham procedures under the same conditions. After birth, the offsprings were assigned to ultrasound-treated or sham groups, to ensure controlled exposure across developmental stages. Three outcome measures were performed: diffusion-weighted MRI, PET imaging and behavioral evaluation using the Morris Water Maze (MWM) test. Previously obtained RNA datasets were subjected to reanalysed to integrate molecular-level alterations with imaging and behavioral findings.

To generate embryos four-week-old female CD1 mice (*n* = 11, 20.17 ± SD grams) purchased from Charles River Laboratories (Sulzfeld, Germany) were housed in individually ventilated cages (Sealsafe Blue line IVC system, Techniplast, Akronom Ltd., Budapest, Hungary) under standard conditions with free access to rodent chow and tap water. They were kept in a facility with a 12-hour light/12-hour dark cycle, at a temperature of 26 ± 2 °C, and 55 ± 10% humidity. Male CD1 mice were sourced from our in-house animal facility and had a mean age of 6 months. Female mice, obtained for breeding purposes, were 3–4 months old at the time of pregnancy, depending on mating conditions. All relevant institutional and national guidelines for animal care and use were followed, and the experiments received approval from the responsible local institutional authority (Ethics Committee for Animal Experimentation of the University of Debrecen, Debrecen, Hungary, license number: 15/2020/DEMÁB) (Fig. 1).

### Ultrasound exposure

Pregnancy timing was determined by the presence of a vaginal plug, which was assessed each morning at 7:30. Plug detection was designated as embryonic day 0.5 (E0.5). Male and female animals were housed in the same cage overnight once a week. Throughout the first (in utero) US exposure, the pregnant mice were anesthetized with Na-pentobarbital at a dose of 50 mg per kilogram of body weight. To ensure that all embryos received an equal US dose prenatally, the transducer was positioned along the midline of the pregnant females, maintaining full contact with the entire abdominal region. The same procedure was used for the control animals, including anesthesia of the pregnant females, handling and physical contact with the transducer, the only difference was that the control animals were not given US exposure. In case of the case of treated animals ten-minute US stimuli were performed in utero and after birth using a GE Vivid E US device (General Electric, Ohio, Cincinnati, USA) with a convex transducer (2.0–5.0 MHz, 128 elements, a convex radius of 60 mm, a field of view (FOV) of 55°, and a footprint of 18.3 × 66.2 mm). US was delivered at a constant frequency of 3.5 MHz, with both mechanical and thermal indexes kept below 1.0 (MI = 0.9; TI = 0.8). Other US output parameters included D = 17, DR = 69 dB, and AO = 100%. The B-mode imaging frequency settings were 2.0, 3.0, 4.0, and 5.0 MHz, while harmonic imaging was performed at 3.0, 4.0, and 5.0 MHz. The calculated peak negative acoustic pressure and the average intensity during exposure were 1.557 MPa and 56 mW/cm^2^, respectively. AquaUltra Basic US gel (Ultragel, Budapest, Hungary) was used to cover the output end of the US probe. In total, 29 treated and 30 control animals were used in our experiment, which involved 5 pregnancies. US exposure was administered once in utero on the last day of gestation (E18.5), and then once per week during the first four postnatal weeks (P7, P14, P21 and P28). After birth, unfocused US was applied postnatally due to the small size of the neonatal skull, and mice were not anesthetized with Na-pentobarbital. We used the same procedure for the control animals, such as handling the pups and physical contact with the transducer. The handling of the treated and control animals was carried out by the same person throughout the experiment so an adaptation (training) of the mice can be expected. The analysis was restricted to female animals (Fig. 1).

### Radiopharmaceutical synthesis

According to the method described by Mikecz et al., [^18^F]2-fluoro-2-deoxy-D-glucose ([^18^F]F-FDG) was produced in the radiochemical laboratory of the Division of Nuclear Medicine and Translational Imaging, Department of Medical Imaging, Faculty of Medicine, University of Debrecen (Debrecen, Hungary) as part of routine synthesis for in-human PET applications^27^. The radiotracer was obtained from a fully automated GE Tracerlab FXFDG module (GE Healthcare, North Richland Hills, TX, USA) in compliance with Good Manufacturing Practice (GMP)^28^.

### In vivo positron emission tomography (PET) imaging

Five mice were randomly selected for brain US exposure one hour after the final treatment, before PET acquisition (US-treated group). The rest of the animals (*n* = 6), receiving no US stimulation, served as controls (control group). All mice (US-treated and control animals) were tail-vein injected with a bolus of 8.24 ± 0.12 MBq of [18 F]F-FDG in 150 µL of physiological saline (Salsol, TEVA, Debrecen, Hungary) under isoflurane anesthesia (1.5% isoflurane, Forane; AbbVie, Budapest, Hungary; OGYI-T-1414/01), with 0.4 L/min oxygen and 1.2 L/min N2O. After a 50-minute wait post-injection, a 20-minute static brain PET scan was performed using the MiniPET II scanner at the preclinical laboratory of the Division of Nuclear Medicine and Translational Imaging, Department of Medical Imaging, Faculty of Medicine, University of Debrecen (Debrecen, Hungary). During PET imaging, inhalation anesthesia was maintained with a dedicated small animal anaesthesia device (Tec3 Isoflurane Vaporizer, Eickemeyer Veterinary Equipment, Luton, UK).

### PET data assessment

Images were reconstructed using the three-dimensional ordered-subsets expectation-maximization line-of-response (3D OSEM-LOR) iterative image reconstruction method. To quantify [18 F]F-FDG uptake, standardized uptake values (SUV) were calculated from volumes of interest (VOIs) that were manually placed over specific brain regions (cerebrum, cerebellum). SUV measurements were performed using the BrainCad image analysis software (University of Debrecen, Debrecen, Hungary) based on the following equation:

$${\mathrm{SUV}} = {{[{\mathrm{VOI}}\;{\mathrm{activity}}\;({\mathrm{Bq}}/{\mathrm{mL}})]} \mathord{\left/ {\vphantom {{[{\mathrm{VOI}}\;{\mathrm{activity}}\;({\mathrm{Bq}}/{\mathrm{mL}})]} {[{\mathrm{injected}}\;{\mathrm{activity}}\;({\mathrm{Bq}})/{\text{animal weight}}\;({\mathrm{g}})].}}} \right. \kern-\nulldelimiterspace} {[{\mathrm{injected}}\;{\mathrm{activity}}\;({\mathrm{Bq}})/{\text{animal weight}}\;({\mathrm{g}})].}}$$

### Morris water maze (MWM) test protocol

Spatial learning was evaluated using the MWM test (8 US-treated and 8 control animals, the same litter was used for PET imaging), following the protocol specified in reference, the age of the animals was P28 at the start of the test^29^. Briefly, a circular water tank approximately 150 cm in diameter and 50 cm high was filled with room temperature water to a depth of 30 cm and placed in a small, quiet, dimly lit room. The tank was divided into four quadrants, with geometric shapes representing the four cardinal directions affixed to the walls as visual cues. A circular platform 10 cm in diameter, mounted on a metal base, was positioned 1 cm below the water surface in the center of one quadrant. To hide the platform from the animals, non-toxic white paint was added to make the water opaque.

The hidden platform remained in the same quadrant throughout both the learning and testing phases, allowing the mice to memorize its location. To improve visibility, all animals were marked with black hair dye around the neck. The training period for platform localization lasted five days. On day 0 (Habituation), the animals were introduced to swimming and the platform. Each session lasted 60 s per animal. During the Testing Phase (Days 1–3), the animals underwent three trials each day for three consecutive days. In this phase, mice were gently placed into the water and given 90 s to find the hidden platform. Because the platform was beneath the opaque water, animals relied on visual cues for navigation. If the animals failed to locate the platform within the allotted time, they were gently guided by applying light pressure at the base of their tails to assist in orientation. A 60-second rest on the platform was provided between trials. The time taken to reach the platform was recorded with a stopwatch, and the data was stored electronically. The average of the three trials per day was calculated, and comparisons across days were made to assess learning ability. On day four (Quadrant Time Phase), the platform was removed, and animals were given a single 45-second trial during which they swam freely. The time spent in the quadrant where the platform was previously located served as a measure of memory retention and was recorded for further analysis. Throughout the experiment, the swimming behaviors of the animals were tracked via video recording.

### Ex vivo diffusion-weighted MRI acquisition and analysis

For MR acquisition, one hour after the last US exposure (P28) the anaesthesia for the autopsy was with sodium pentobarbital (Nembutal, 50 mg/kg), after the deep anaesthesia, mice were transcardially perfused with 0.9% NaCl saline followed by 4% PFA (buffered to pH 7.6) perfusion, followed by a saline flush. The brains were removed from the skull and the whole brain was further fixed with immersion into 4%PFA for overnight at 4 °C and kept in PBS until they were being imaged. For DTI-based MR examinations, two separate groups were formed, each with 7 animals: US-treated and control. Following post-fixation, the brains were rinsed in PBS and positioned in a custom-designed 3D holder within 5 mL Eppendorf tubes. To ensure a uniform, black background and reduce susceptibility artifacts during imaging, the tubes were filled with Fluorinert™ (Fluorinert FC-40, Sigma-Aldrich). Each brain specimen was placed into an ex vivo scanner holder for imaging. A total of 14 brains (7 treated, 7 control) were scanned using a 7-T Bruker BioSpec 70/30 MRI system (Ettlingen, Germany) with a maximum gradient strength of 680 mT/m, but we lost one sample from the control group. T2-weighted (T2W) images were acquired using a RARE (Rapid Acquisition with Refocused Echoes) sequence with the following parameters: matrix size = 256 × 256, slice thickness = 700 μm, repetition time (TR) = 2500 ms, and echo time (TE) = 11 ms. Diffusion-weighted MRI (DW-MRI) was performed on the same subset of ex vivo brain samples using a stimulated echo planar imaging (EPI) sequence. The acquisition protocol included 125 diffusion gradient directions with b-values of 0 s/mm^2^ (*n* = 5), 4000 s/mm^2^ (*n* = 60), and 7000 s/mm^2^ (*n* = 60). Additional imaging parameters were: diffusion time = 15 ms, diffusion gradient duration = 5.5 ms, TR = 5000 ms, and TE = 28 ms. Forty slices covering the entire brain were obtained with a field of view (FOV) of 18 × 15 mm^2^ and a matrix size of 120 × 100, resulting in an in-plane resolution of 0.15 × 0.15 mm^2^. Each slice was 0.6 mm thick, and data acquisition was averaged over 7 signal repetitions. DW-MRI data were corrected for eddy current distortions, and brain extraction was performed using the SAMson toolbox^30^. Diffusion tensor fitting was conducted using FSL’s dtifit. Tract-Based Spatial Statistics (TBSS) analysis from FSL was then applied to compare diffusion metrics between the control and treated groups (*n* = 7 per group). Additionally, region-of-interest (ROI) analyses of fractional anisotropy (FA) and mean diffusivity (MD) maps using Fiji were performed. For each hemisphere, a single ROI measuring 2 × 2 voxels was manually placed. The corpus callosum and fimbriae were specifically analysed by placing two ROIs per brain structure and subject. Group-level averages were calculated for each structure, and statistical comparisons between conditions were performed using two-sample t-tests.

### Total RNA preparation and quality assessment for RNA seq analysis

We used the same RNA-seq dataset as earlier described (Winkler et al. 2024) and re-analyzed it, the KEGG pathways presented here are derived from the same differentially expressed gene list reported previously^7,31^. The raw data is available (http://www.ncbi.nlm.nih.gov/bioproject/PRJNA1058398).

Briefly, one hour after the last US treatment, 9 control and 9 US-treated mice (P28) were sacrificed with sodium pentobarbital (150 mg/kg). We isolated both hippocampi from the animals without separating the left and right sides. Following hippocampal preparation, the samples were homogenised and immersed in TRIzol (Ambion, Life Technologies, CA, USA). After RNA extraction, RNA concentration and A260/280 ratio were measured using a spectrophotometer (DeNovix, Inc., Wilmington, DE, USA), and following quality control, 10 µg of RNA from each sample were pooled. Then, the quality of the RNA samples was assessed using an Agilent BioAnalyzer (Agilent Technologies, Inc., Santa Clara, CA, USA) with the Eukaryotic Total RNA Nano Kit, following the manufacturer’s protocol. Samples with an RNA integrity number (RIN) of 7 or higher were selected for library preparation. To obtain global transcriptome data, high-throughput mRNA sequencing was performed using the Illumina sequencing platform (Illumina, Inc., San Diego, CA, USA). RNA-Seq libraries were prepared from total RNA using the Ultra II RNA Sample Prep kit (New England Biolabs) according to the manufacturer’s instructions. Poly-A RNAs were captured with oligo-dT conjugated magnetic beads, then eluted, and the mRNAs were fragmented at 94 °C. First-strand cDNA was synthesized using random priming reverse transcription, followed by second-strand synthesis to generate double-stranded cDNA. The cDNA was repaired, A-tailed, and adapter-ligated. Adapter- ligated fragments were amplified via enrichment PCR, and sequencing libraries were finalized. Sequencing runs were conducted on an Illumina NextSeq 500 using single-end 75-cycle sequencing. The raw sequencing data (FASTQ files) were aligned to the mouse reference genome version MM10 using HISAT2, resulting in BAM files. Downstream analysis was performed using StrandNGS software (http://www.strand-ngs.com). BAM files were loaded into the analysis software, and normalization was carried out using the DESeq algorithm. Network and functional enrichment analyses were conducted with Cytoscape (ver 3.10.1), and gene ontology (GO) biological processes were mapped using the EnrichmentMap app (ver 3.3.6).

### Statistical analysis

Data presented are the results of at least three independent series of measurements expressed as mean ± SD. For the analyses of DTI data two-sample t-test, for PET data assessment Mann-Whitney were used, and for all statistical calculations we applied the commercial software package MedCalc 18.5 (MedCalc Software, Mariakerke, Belgium). RNA seq data were analysed using *Z*-test with Benjamini–Hochberg FDR to determine the differentially expressed genes between conditions. Behavioral data obtained from the Morris Water Maze test were assessed with repeated-measures analysis of variance (two-way RM-ANOVA) with Geisser–Greenhouse correction, followed by Tukey’s post hoc test; in the case of the quadrant time, we used an unpaired t-test implemented in GraphPad Prism (version 9.0; GraphPad Software Inc., La Jolla, CA, USA). The significance level was set at *p* < 0.05. The figures were generated using Microsoft Excel (Microsoft 365; Microsoft Corporation, Redmond, WA, USA) and subsequently edited in GIMP (GNU Image Manipulation Program) for final layout and formatting (Fig. 1).

## Results

### [^18^F]FDG PET results

**Fig. 2:**
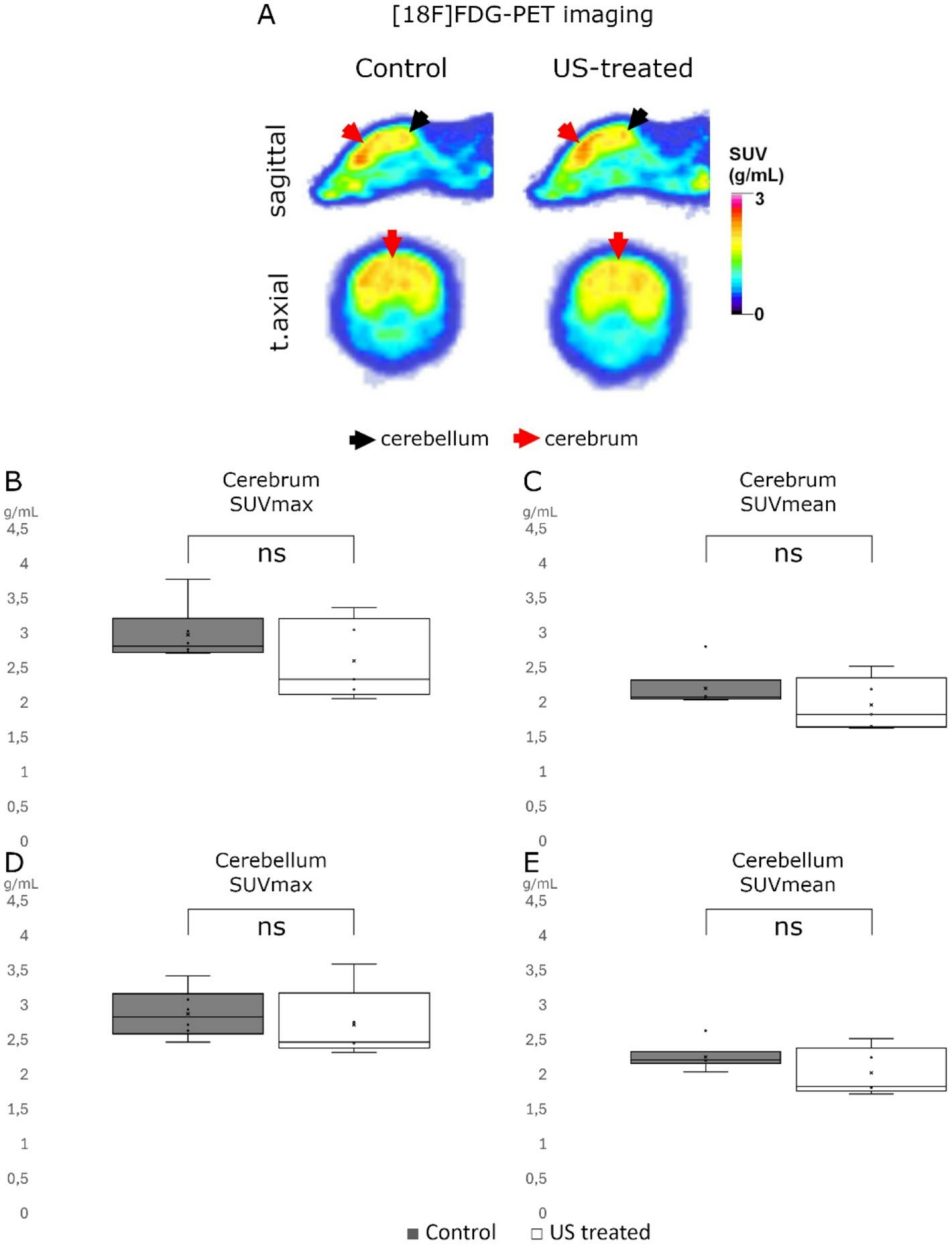
**A** Representative sagittal (upper row) and axial (lower row) [^18^F]FDG PET images of a control (left) and a US-treated (right) study animal. Box-and-whiskers plots demonstrate the [^18^F]FDG radiotracer uptake of the cerebrum (**B** (SUVmax ) and **C** (SUVmean)) and cerebellum (**D** (SUVmax) and **E** (SUVmean)) of the control (grey) and US-treated (white) groups. No statistically significant differences were found between the radiopharmaceutical uptake of the examined groups. Box plots show median, quartiles, and range, and individual data points.

US-treated and control mice underwent [18F]FDG PET scans to assess possible metabolic changes caused by US exposure. Visual inspection of brain regions in the cerebrum and cerebellum revealed similar radiotracer uptake levels between the US-stimulated and control groups (Fig. 2, Panel A). Quantitative analysis of PET data confirmed this, showing no statistically significant differences in radiopharmaceutical accumulation either in the cerebrum (Fig. 2, Panels B and C) or the cerebellum (Fig. 2, Panels D and E). Specifically, cerebral SUVmean was 2.19 ± 0.30 in controls and 1.96 ± 0.38 in treated mice; cerebral SUVmax was 2.97 ± 0.41 in controls and 2.59 ± 0.58 in treated mice; cerebellar SUVmean was 2.25 ± 0.20 in controls and 2.02 ± 0.32 in treated mice; and cerebellar SUVmax was 2.87 ± 0.34 in controls and 2.71 ± 0.51 in treated mice. The comparison of [18F]FDG uptake in the selected brain areas of both groups is shown in (Fig. 2).

### MWM test results

**Fig. 3:**
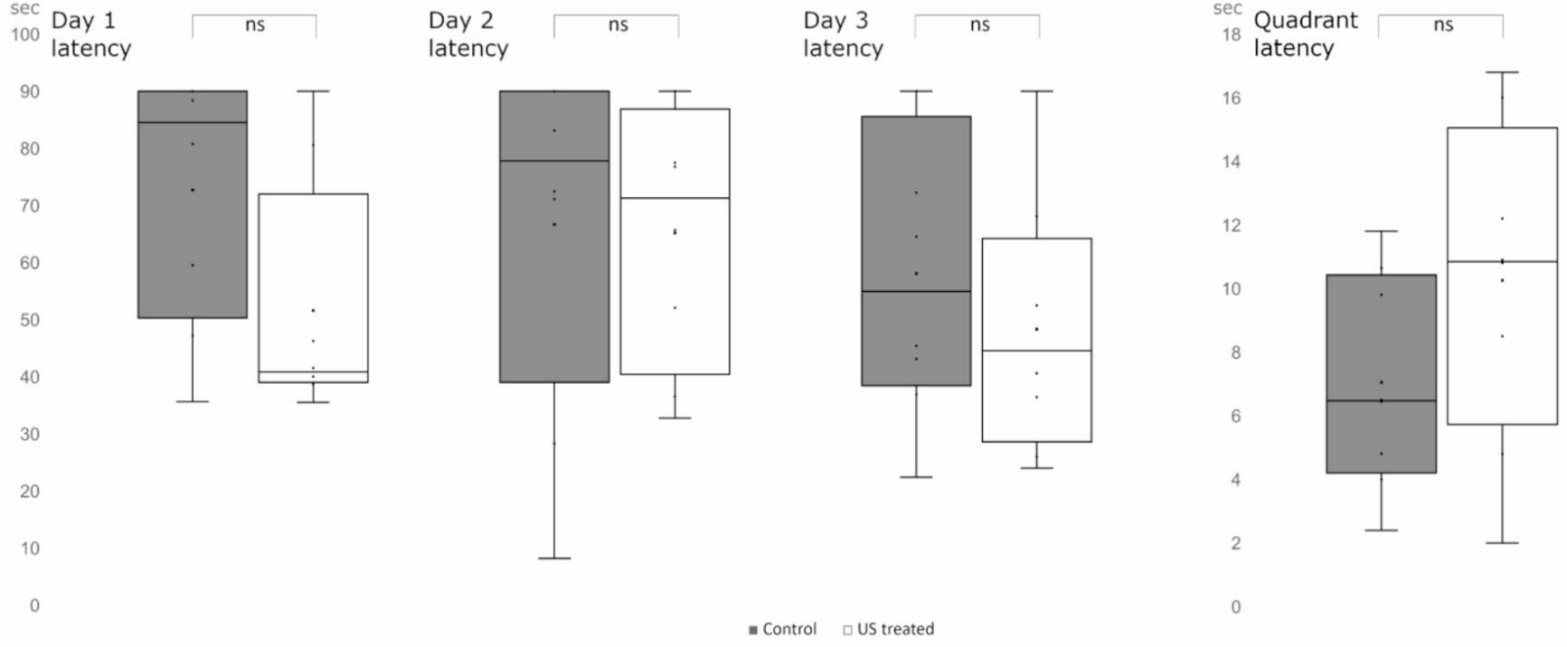
Box-and whiskers plots showing the results of MWM test in the US-treated (white) and the control (grey) groups. MWM test of day 1 (control: 72.68 ± 22.05 s, US-treated: 51.58 ± 21.17 s), day 2 (control: 66.63 ± 31.28 s, US-treated: 65.16 ± 22.55 s), day 3 (control: 58.05 ± 25.04 s, US-treated: 48.25 ± 22.15 s, ​), time in target quadrant (control: 7.04 ± 3.37 s, US-treated: 10.25 ± 5.08 s). Box plots show median, quartiles, and range, and individual data points. Significance level: *p ≤ 0.05. ​.

To investigate the impact of US exposure on memory and cognitive performance, 8–8 mice (both the US-treated and control groups) were subjected to the MWM test ((data analysis: Two-way RM ANOVA and unpaired t-test (*p* = 0.05)). As shown in Fig. 3, no significant changes were observed between the US-treated and the control groups (day x treatment *p* = 0.34, degrees of freedom: 2, F values: 1.10; time: *p* = 0.16, degrees of freedom: 2, F values: 1.95; treatment: *p* = 0.27, degrees of freedom: 1, F values: 1.30; individual: *p* = 0.005, degrees of freedom: 14, F values: 3.04). There were no significant between-group differences on day 1 (control: 72.68 ± 22.05 s, US-treated: 51.58 ± 21.17 s) days 2 and 3 (day 2 control: 66.63 ± 31.28 s, day 2 US-treated: 65.16 ± 22.55 s, day 3 control: 58.05 ± 25.04 s, day 3 US-treated: 48.25 ± 22.15 s). Identically, no considerable differences were found in quadrant time between the US-treated and the untreated, control mice (control: 7.04 ± 3.37 s, US-treated: 10.25 ± 5.08 s, *p* = 0.16).

### Hippocampal formation RNA-seq analysis

**Fig. 4:**
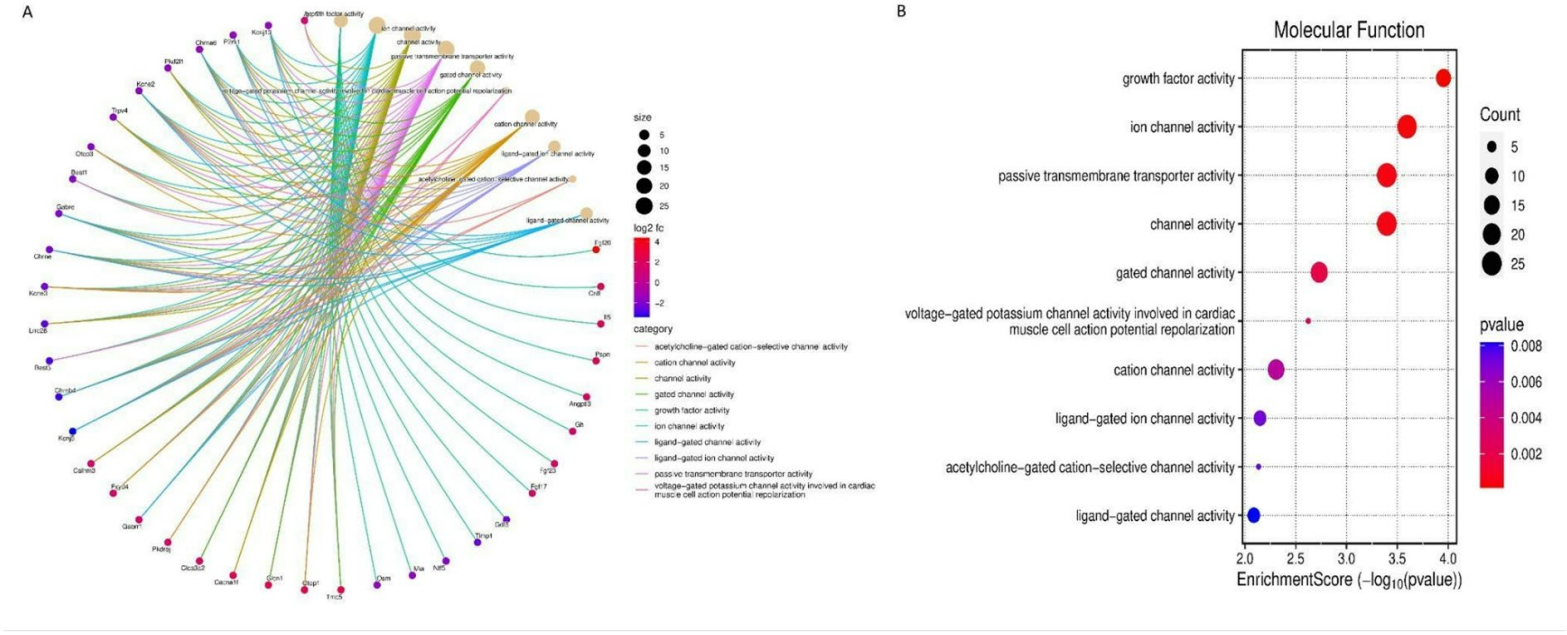
Comprehensive RNA-seq analysis and GO biological processes. The figure is divided representing different aspects of the RNA-seq differential expression (DE) analysis. The panels were generated by the EnrichmentMap (ver 3.3.6) based on the KEGG database criteria^31^. The size of each bubble corresponds to the number of genes involved in each process, while the color indicates the *p*-value (log10 value). Panel (**A**) shows putative protein-receptor interactions related to calcium signalling, while Panel (**B**) indicates the strength of the molecular function alteration between the samples.

The results of the pathway analysis on our bulk RNA-seq data comparing US-treated and control samples are shown in Fig. 4. Regarding calcium signaling, we found a significant difference between the samples (pathway ID: mmu04020, gene ratio: 11/205, *p* value: 0.0206, genes analysed included Fgf20, Cacna1f, Bdkrb2, Agtr1a, Fgf23, Fgf17, Ltb4r2, Atp2a1, Trhr2, Calml4, P2rx1). In contrast, for the long-term potential pathway (ID: mmu04720), no significant changes were observed (gene ratio: 1/205, *p* value: 0.78), and in this case, only the Calml4 protein was analysed. Using EnrichmentMap (ver 3.3.6), our analysis identified several potential molecular functions related to a broad range of channel activities, as shown in Fig. 4.

### Microstructural alterations in the brain parenchyma

**Fig. 5:**
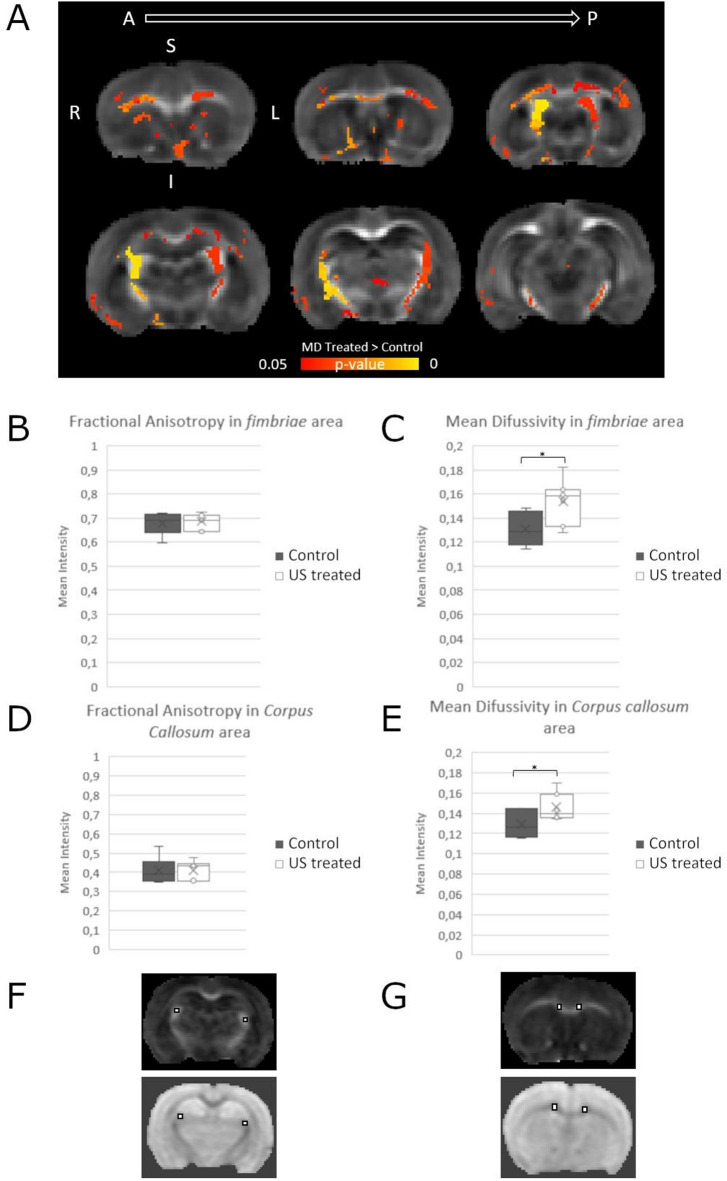
Microstructural assessment of US-treated and control mouse brains using ex vivo diffusion MRI. (**A**) TBSS voxel-wise comparison of mean diffusivity (MD) overlaid on the mean fractional anisotropy (FA) shows significantly increased MD in treated mice compared to controls (*p* < 0.05; *n* = 7 treated, *n* = 6 control). (**B**–**E**) Box-and-whisker plots comparing MD and FA values between treated and control groups. In the fimbria we measured the FA (**B**) and MD (**C**), we also analysed the FA in the corpus callosum (**D**), and the MD (**E**). Group means were compared using two-sample t-tests. Data are shown as median (line), mean (cross), interquartile range, and full data range. (**F**–**G**) Representative regions of interest (ROIs) used for quantification in the *fimbria* (FA map and MD map) (**F**) and *corpus callosum* (FA map and MD map) (**G**) (2 × 2 voxels per hemisphere).​

Ex vivo diffusion MRI analysis revealed significant microstructural differences in white matter regions between the US-treated and control mice. Voxel-wise comparisons (TBSS, see methods) showed a notable increase in MD in the treated group compared to controls (Fig. 5, *p* < 0.05; *n* = 7 treated, *n* = 6 control animals). Region-of-interest (ROI) analyses further confirmed these findings, indicating significantly higher MD values in the fimbria and corpus callosum of the treated mice (Fig. 5B and D). No significant differences were observed for FA (Fig. 5C and E). Overall, these results suggest that repeated prenatal and postnatal US exposure may cause subtle microstructural changes in the major white matter tracts of the developing brain.

## Discussion

Based on current evidence, using sonography during pregnancy has not been linked to adverse maternal or perinatal outcomes, nor has it been associated with disruptions in normal physical or neurological development, or with an increased incidence of cancer, intellectual impairment, or mental illness in humans^1^. Consistent with these findings, results from our preclinical experiment indicate that repeated diagnostic-level US exposure do not influence the memory function, but modify white matter connectivity in the central nervous system as well as the RNA expression profile, without affecting brain glucose metabolism.

The experimental design employs the Clancy–Workman scaling framework to convert murine ultrasound exposure windows to specific human neurodevelopmental stages^32^. Initial exposure at E18.5 corresponds to the human late second trimester (gestational weeks 22–26), which is a critical phase involving active neuronal migration, cortical lamination, and the establishment of thalamocortical connections, this phase occurs shortly after the standard mid-gestation anomaly scan^33^. Postnatal murine developmental stages have been shown to correspond to the continuum of human brain maturation: P7 is comparable to the late preterm period (28–34 weeks of gestation), which is characterised by accelerated synaptogenesis^34^. P14 mirrors the human term and neonatal period, which is characterized by synaptic refinement. Subsequent exposures (P21 and P28) correspond to early infancy (ages 2–6 months), encompassing dendritic maturation and the onset of myelination^35^.

### Effects of multiple applications of the same diagnostic dose US on the memory of model animals 

Recent data show that repeatedly used diagnostic US exposure significantly affects various morphological characteristics of the central nervous system, such as the dendritic tree density of excitatory neurons, the spine morphology of CA1 neurons, and the development of atrophic changes within the hippocampal complex^7,20,36^. The hippocampus mainly functions in memory formation, retrieval, and spatial processing, with theta wave activity - commonly observed during REM sleep — playing a key role in integrating memories^37–39^.

Previous studies have reported that US exposure alters cognitive function; however, our results show no significant effect on cognitive performance when the same diagnostic US level is used repeatedly^40–42^. Earlier findings of a prior study indicated that the FDA-approved US dose (4 min with TI and MI below 1 and FR of 3.5 MHz) enhances memory abilities in rat pups^40^. In contrast, other studies using high-intensity US exposure (20 min, MI: 1.4, TI: 1, 3.5 MHz) reported impairments in learning and memory, which were closely linked to reductions in BDNF and NMDA receptor expression. Moreover, this research suggests that abnormal layering of the neocortex and hippocampal cortex, along with destruction of synaptic structures caused by extensive US radiation, may also contribute to altered memory capabilities^39,40,43^. In the study by Suresh et al., US exposure in rats at E14 with similar parameters to ours (3.5 MHz, 65 mW/cm2) but longer duration (30 min) impaired the memory, while 16-day-old embryos showed relative resilience to the same US exposure in adulthood (4-months-old animals)^42^. Notably, histological analysis revealed lower neurotransmitter levels and reduced hippocampal neuronal density in the E14 group, highlighting the critical influence of exposure time and duration^42^. Furthermore, analysing the social behavior of pups exposed to US at E14.5, McClintic and colleagues observed significantly reduced social interaction interest in the treated group compared to controls (*p* < 0.01) three weeks after birth^44^. Additionally, although the exact mechanism remains unclear, treated animals exhibited significantly increased activity levels compared to sham-exposed offspring (*p* < 0.05), but only in the presence of an unfamiliar conspecific^44^.

According to our result the PET examinations did not show any differences between the cerebral metabolism of the US-treated and control groups. To the best of our knowledge, this is the first study to evaluate metabolic changes induced by US signaling in the developing brain; therefore, a detailed understanding of the current findings remains for future research. Even so, we believe that metabolic changes may take more time to become apparent, and imaging at later or multiple time points could offer a more complete assessment of US-induced impairments in brain glucose homeostasis ([^18^F]FDG uptake) in utero.

### Calcium signaling and long-term potentiation (LTP)

Literature data indicate that US waves exert their cellular effects through multiple mechanisms, including direct damage to cell membranes and organelles, as well as activation of various mechanosensitive channels such as Piezo1, TRPA1, TRPC4, TRPV4, MEC-4, and voltage-gated Na + and Ca2 + channels^45–50^. Although many questions remain about how US-induced activation of these ion channels leads to dendritic growth, an increase in intracellular Ca2 + levels mediated by downstream MAPK or ERK1/2 signaling pathways through these mechanosensitive ion channels (such as TRPV4 and Piezo1), as well as glutamatergic (NMDA, AMPA) receptors, may play a key role in this process^46^. Intracellular Ca2 + signaling is essential for inducing LTP, which forms the basis of long-term memory formation^51^. As the principal cation regulating both the magnitude and duration of LTP, a cellular increase in Ca2 + concentrations is crucial for modulating the expression levels of neurotrophic factors like BDNF^52^. This aligns with our findings, which also showed elevated BDNF levels following US stimuli. Since NMDA receptors mediate Ca2 + influx and subsequent LTP, our observation of BDNF-related upregulation of NMDA receptor subunits, including NR1, NR2A, and NR2B, further confirms the connection between BDNF expression and rising Ca2 + levels. Additionally, the opening probability of NMDA receptors is regulated by Ca2+, indicating activity-dependent feedback inhibition and Ca2+-dependent receptor inactivation^53,54^. Similar to our current findings, elevated BDNF levels triggered by US signaling were reported by Papp et al.^6^ and Tufail and colleagues^55^. Intriguingly, and unlike our observations, the study by Papp and co-workers^6^ found no US-related changes in BDNF gene expression as assessed by qPCR.

### US-induced changes in CNS connectome

To the best of our knowledge, this is the first publication to report changes in the CNS connectome related to US exposure. Considering the important role of BDNF in regulating myelination, we hypothesize that the white matter changes observed after US treatment may be due to increased BDNF protein levels. Earlier studies also linked BDNF to CNS regeneration by promoting oligodendrocyte maturation and myelin sheet formation, which supports our observations and hypothesis^56–58^. Similarly, elevated BDNF levels induced by US signaling have been identified as a potential protective factor that enhances neuronal activity and promotes regeneration in demyelinating diseases such as multiple sclerosis (MS)^56–58^. According to the literature, BDNF’s diverse functions enable endogenous repair mechanisms, regeneration, and remyelination after demyelinating inflammatory injury in MS, providing a basis for neuronal preservation and slowing disease progression^56–58^. It is noteworthy that, although BDNF has a well-established role in myelination, the valine (Val) to methionine (Met) change in the 5’ pro-region of the protein (Val66Met polymorphism)^59^ influences its neurological effects and the structure of brain regions involved in learning and memory, such as the hippocampus and prefrontal cortex^59^. Therefore, potential BDNF variations should be considered when evaluating US-induced changes in the CNS connectome. Additionally, besides BDNF, cognitive training has been shown to influence structural connectivity^60^. Finally, our findings of altered white matter connectivity in the fornix following US exposure may align with prior studies documenting US-associated changes in θ oscillations within this region^23,26^.

### Limitations

Although several biological processes are conserved across species, significant differences between rodent and human brains present challenges for directly applying our findings to humans. Because of the larger relative area exposed to the US field and the thinner skull, rodent brains are more vulnerable to US exposure than human brains. The physical stimuli may cause behavioural changes, however, both the treated and control groups received identical handling, suggesting that stress may have affect both groups equally. Also, the current US treatment lasted much shorter than human US procedures, which could further complicate translating these preclinical results to humans. It is important to note that these were B-mode ultrasound exposures; in human practice, color Doppler and power Doppler type US examinations are also used, which increase the amount of energy delivered. Addressing these differences between rodents and humans remains a focus for future research.

## Data Availability

The original contributions presented in the study are included in the article/supplementary material; further inquiries can be directed to the corresponding author/s. The RNAseq raw data is available: http://www.ncbi.nlm.nih.gov/bioproject/PRJNA1058398.
